# Quantification of SARM1 NADase Activity in Human Peripheral Blood Mononuclear Cells

**DOI:** 10.1096/fj.202504642R

**Published:** 2026-05-02

**Authors:** Lila F. Dabill, Ivana R. Shen, Jennifer M. Brazill, Alicia Neiner, Yo Sasaki, Erica L. Scheller

**Affiliations:** ^1^ Division of Bone and Mineral Diseases Washington University School of Medicine St. Louis Missouri USA; ^2^ Department of Biomedical Engineering Washington University in St. Louis St. Louis Missouri USA; ^3^ Department of Genetics Washington University School of Medicine St. Louis Missouri USA; ^4^ Department of Developmental Biology Washington University School of Medicine St. Louis Missouri USA

## Abstract

SARM1 (sterile *α* and TIR motif‐containing protein‐1) is an NADase enzyme that serves as the central executioner of Wallerian axon degeneration. Given this, SARM1 is of high interest as a candidate therapeutic target, and SARM1 inhibitors are currently in clinical trials for treatment of neurodegeneration. Beyond neuroscience, emerging studies reveal that SARM1 may also drive aspects of bone fragility, liver pathology, adipose expansion, and insulin resistance in metabolic disease. However, we lack methods to quantify SARM1 activation in humans to better define patients at high risk of SARM1‐mediated tissue damage. Unlike neurons, peripheral blood mononuclear cells (PBMCs) represent an easily accessible population for clinical screening. While SARM1 gene expression has been identified in PBMCs, it is less known whether functional SARM1 NADase is present. We hypothesized that by pairing activators and inhibitors of SARM1 with analysis of downstream changes in cellular metabolites, we could identify and quantify both basal SARM1 activity and the SARM1 activation potential of human PBMCs. Our results reveal that SARM1 agonist pyrinuron, also known as Vacor, activates a dose‐dependent increase in cAPDR and the cADPR:ADPR ratio that is arrested when paired with SARM1 inhibitor DSRM‐3716. Changes in secondary metabolites including NAD+, NMN, NaMN, ATP, AMP, IMP, inosine, and succinyl adenosine were also characterized and used to generate a working model of PBMC SARM1 activation. Overall, these findings demonstrate that human PBMCs have detectable SARM1 activation potential and could be leveraged as a clinical readout of SARM1 expression and activity across diverse disease contexts.

## Graphical abstract

Proposed model for NAD+ metabolism in (A) control and (B) SARM1 activated PBMCs. In native form, 3‐AP and Vacor compete with NAM as an alternative substrate for NAMPT. This consequently decreases generation of NMN and biosynthesis of NAD+ is limited by both a reduction in precursor NMN and direct inhibition of NMNAT by Vacor‐MN and 3‐AP‐MN. Vacor‐MN and 3‐AP‐MN also increase NAD+ consumption by activating SARM1. Addition of orthostatic SARM1 inhibitor DSRM‐3716 blocks NAD+ consumption by SARM1 but does not prevent the decrease in NAD+ biosynthesis. We hypothesize that most cellular ADPR in PBMCs is generated by other highly expressed hematopoietic NADases such as CD38. Activation of SARM1 in PBMCs appears to preferentially convert NAD+ to cADPR, as reported previously in neurons. We propose that depletion of the NAD+ pool and SARM1‐dependent generation of cADPR limits the availability of substrate for alternative NADase‐based generation of ADPR, resulting in decrease.

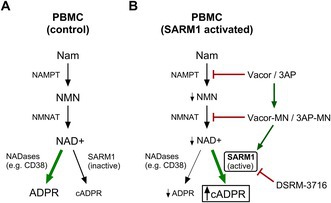

## 1 Introduction

SARM1 (sterile *α* and TIR motif‐containing protein‐1) is an evolutionarily conserved NADase that is most highly expressed in the nervous system. SARM1 serves as the central executioner of Wallerian axon degeneration and can be activated in response to injury, inflammation, and oxidative stress [1, 2, 3, 4]. Based on this, SARM1 inhibitors are currently in Phase I clinical trials for the prevention and treatment of neurodegenerative diseases [5, 6, 7]. To support the success of these therapies, it is important to consider how blocking SARM1 NADase function might affect other cell types to better predict and understand clinical outcomes. There is also a need to develop assays that can be used in humans to track activation of the SARM1 NADase.

Multiple RNAseq atlases demonstrate low but detectable expression of SARM1 in non‐neural tissues [5, 6]. However, the specificity and functional significance of low SARM1 expression at these sites, if any, remain unclear. To date, the best evidence for non‐neural actions of SARM1 relates to its expression in hematopoietic lineage cells, as reported in 13 publications since 2006 [7, 8, 9, 10, 11, 12, 13, 14, 15, 16, 17, 18, 19]. Early conclusions were confounded by nonspecific antibodies [8] and unanticipated passenger mutations in the original Sarm1 knockout mouse [14, 20]. However, follow‐up work using RT‐PCR, qPCR, northern blot, SARM1 GFP reporter, and FLAG‐tagged SARM1 protein provides a consensus that SARM1 is expressed, albeit at low levels, in hematopoietic lineages, including monocytes, macrophages, and lymphocytes [7, 8, 9, 10, 11, 12, 13, 14, 15, 16, 17, 18, 19, 21, 22, 23, 24, 25]. Consistent with this, 6 prior reports have confirmed detectable but low *SARM1* gene expression in human peripheral blood mononuclear cells (PBMCs) [7, 8, 9, 13, 15, 17, 25].

PBMCs represent a diverse and easily accessible population for clinical screening and play a critical role in inflammation and immunity. However, an important gap in knowledge is whether primary human PBMCs have basal, endogenous SARM1 NADase activity or if SARM1 NADase can be cell‐autonomously activated in these populations. To overcome this while supporting the development of SARM1‐targeting therapies, we hypothesized that by pairing activators and inhibitors of SARM1 with analysis of downstream changes in cellular metabolites by mass spectrometry, we could rigorously quantify both the basal SARM1 activity and the SARM1 activation potential of primary human PBMCs.

Activation of the SARM1 NADase occurs in response to injury, inflammation, and oxidative stress due to a high ratio of nicotinamide mononucleotide (NMN) to nicotinamide adenine dinucleotide (NAD+) within the cell and binding of NMN to the allosteric activation site of SARM1 [26]. This can be bypassed through direct SARM1 activation using either 3‐acetylpyridine (3‐AP) or pyrinuron, also known as Vacor, which are converted to 3‐AP‐MN and Vacor‐MN by nicotinamide phosphoribosyltransferase (NAMPT) and bind SARM1 in lieu of NMN [27, 28]. The enzymatic cleavage of NAD^+^ through activated SARM1 subsequently creates byproducts, including nicotinamide (NAM), cyclic ADP‐ribose (cADPR), and ADP‐ribose (ADPR), with previous work in neurons showing that cADPR accumulation is a sensitive and specific readout of SARM1 activity [29].

To test this in PBMCs, we performed functional assays of SARM1 using known inhibitors and activators of its enzymatic activity [27, 28, 30]. Specifically, human PBMCs were stimulated with SARM1 agonists 3‐AP (1 mM) and Vacor (10, 100, 500 μM), for 4 h to activate the enzymatic activity of SARM1. Agonist responses were compared to treatment with vehicle control for each independent participant. To confirm specificity for SARM1, a paired sample of cells within each group was simultaneously treated with 100 μM of SARM1 inhibitor DSRM‐3716, which binds to the orthosteric site of SARM1 to inhibit its NADase activity [30]. Importantly, DSRM‐3716 has been proven to be a highly specific inhibitor of the SARM1 NADase and does not affect the function of other NADases such as CD38 [30]. Our results reveal that Vacor drives a dose‐dependent increase in cAPDR that is arrested when paired with SARM1 inhibitor DSRM‐3716. Changes in secondary metabolites including NAD+, NMN, NaMN, ATP, AMP, IMP, inosine, and succinyl adenosine were also characterized, leading to a proposed working model for SARM1 function within PBMCs as it relates to NAD+ metabolism. Overall, these findings reveal that human PBMCs have detectable SARM1 activation potential and could be leveraged as a clinical readout of SARM1 expression and activity across diverse disease contexts. These methods could also be applied to investigate SARM1 activation potential in other cell types.

## 2 Methods

### 2.1 Human PBMCs

This study included PBMCs isolated from adolescent girls aged 12 to 16 years (*n* = 7, mean 15.1 ± 1.8 years). Informed consent and assent were obtained from the guardian and participants, respectively, under IRB #201908120. Participants were recruited from Washington University. Exclusion criteria included medical conditions, including anemia, osteogenesis imperfecta, fetal alcohol syndrome, leukemia, congenital heart defects, neurological disorders, untreated hyper‐ or hypothyroidism, rheumatoid arthritis, hyperparathyroidism, epilepsy, Parkinson's disease, cancer, coronary artery disease, peripheral vascular insufficiency, stroke, or premature ovarian failure. In addition, subjects using medications known to impact bone metabolism (bisphosphonates, glucocorticoids, GH) were excluded.

### 2.2 RNA Extraction and qPCR

Washed cells were resuspended in 600 μL of lysis buffer with freshly added 1% β‐mercaptoethanol (β‐ME) and homogenized with a 27‐gauge needle. RNA was isolated using the Invitrogen PureLink RNA column (#12183018A) and eluted in 30 μL of nuclease‐free water. RNA samples were stored at −80°C prior to analysis. RNA samples were transcribed to cDNA using the Maxima H Minus cDNA Synthesis kit (Thermo Fisher Scientific, M1682) according to the manufacturer's instructions. TaqMan quantitative PCR was used to measure gene expression levels in the cDNA. TaqMan Fast Advanced 2X Master Mix for qPCR (Applied Biosystems, #4444557). *SARM1* expression was measured using the Hs00248344_m1 primer assay. The expression level was calculated based on a cDNA standard curve and compared to gene expression of ACTB (Hs99999903_m1) and HPRT1 (Hs02800695_m1).

### 2.3 PBMC Purification and Treatment

Whole blood was collected from volunteer participants into three 6 mL K2 EDTA‐treated vacutainers. The tubes were inverted multiple times to ensure complete mixing of the anticoagulant with the blood. Vacutainers were transported on ice and processed within 1 h of collection. Approximately 15 mL of anticoagulant‐treated blood was transferred to a 50 mL conical tube and diluted with 15 mL of room‐temperature Dulbecco's Phosphate Buffered Saline (DPBS). Separately, Ficoll‐Paque media was mixed thoroughly by inverting the container. A 15 mL aliquot of the Ficoll‐Paque media was withdrawn using a syringe and added to a new 50 mL conical tube. The diluted blood (30 mL) was gently layered onto the Ficoll‐Paque media without mixing. The conical tube was centrifuged at 400 × g for 30 min at 18°C with no brake applied. This process resulted in distinct layers, including the plasma layer on top, the mononuclear cell layer in the middle, and Ficoll‐Paque and red blood cells at the bottom. The plasma layer was carefully aspirated till the mononuclear cell layer. The mononuclear cells were then collected using a sterile Pasteur pipette, ensuring minimal contamination with Ficoll‐Paque media or the remaining plasma. The collected mononuclear cells were transferred to a new 50 mL conical tube and washed with 30 mL of DPBS. The tube was centrifuged at 150 × g for 10 min at 18°C. After centrifugation, the supernatant was removed, and the cell pellet was resuspended in complete RPMI 1640 media supplemented with 10% patient‐derived serum to achieve a final density of approximately 2 × 10^6^ cells/mL. Cells were divided into tubes for RNA isolation and metabolite extraction based on the number of treatment conditions. For example, if there were five treatment groups (Basal Condition, Vehicle, 10 μM DSRM‐3716, 1 mM 3‐AP, and 10 μM DSRM‐3716 + 1 mM 3‐AP), the cells were divided into ten 1.5 mL tubes, each containing approximately 2 million cells. The number of tubes and cell volumes were adjusted according to the total yield from the PBMC isolation process.

### 2.4 Metabolite Extraction

Washed cells were lysed in 160 μL ice‐cold 50% MS grade MeOH in H_2_O, which was mixed thoroughly and incubated on ice for 5 min to extract metabolites. The 1.5‐mL microcentrifuge tubes were centrifuged at 2000 × g at 4°C for 5 min to pellet debris. The supernatant containing extracted metabolites was transferred to a new tube and mixed vigorously with 50 μL of HPLC‐grade chloroform. The tubes were centrifuged at 20000 × g at 4°C for 15 min. The clear upper aqueous phase was collected and stored at −80° *C. prior* to LC–MS analysis, the samples were thawed and lyophilized under vacuum.

### 2.5 Metabolite Measurement Using LC–MS/MS

Lyophilized samples were reconstituted with 70 μL of 5 mM ammonium formate and centrifuged at 12000 × g for 10 min. The cleared supernatant was transferred to sample vials. Serial dilutions of standards for each metabolite in 5 mM ammonium formate were used for calibration. HPLC‐mass spectrometry analysis was performed on an Agilent 1290 Infinity II liquid chromatography system (Agilent Technologies, Santa Clara, CA) with a flexible pump, multisampler, sample cooler, and an MCT containing an Atlantis T3 column (2.1 × 150 mm, 3 μm) and VanGuard guard cartridge (2.1 mm × 5 mm, 3 μm) (Waters, Milford, MA), coupled to an Agilent 6470 Triple Quad mass spectrometer (Agilent Technologies, Santa Clara, CA). The mobile phase (0.15 mL/min) was 5 mM ammonium formate in water (A) and 100% methanol (B). The column was equilibrated with 0% B, maintained after injection for 2 min, then a linear gradient to 20% B applied over 4 min. The column was then ramped to 50% B over 2 min, and held at 50% for 2 min, then reverted back to 0% B over the next 5 min and allowed to re‐equilibrate at 0% B for 9 min. The total run time was 24 min per sample. The injection volume was 10 μL. The mass spectrometer was equipped with an electrospray ion source which was operated in positive ion multiple reaction monitoring (MRM) mode for the detection of all metabolites. The [M + H] + transitions were optimized for each metabolite and were selected as follows: m/z 560 → 136 for ADPR, m/z 348 → 136 for AMP, m/z 508 → 136 for ATP, m/z 542 → 428 for cADPR, m/z 349 → 137 for IMP, m/z 269 → 137 for Inosine, m/z 664 → 428 for NAD, m/z 123 → 80 for Nam, m/z 336 → 124 for NaMN, m/z 335 → 123 for NMN, and m/z 384 → 252 for s‐AD. The mass spectrometer settings for the fragmentation, the collision energy (CE) and the cell accelerator voltage were optimized for each of these transitions. Raw data were acquired and quantified using MassHunter Workstation software version B.08.00 for 6400 Series Triple Quadrupole (Agilent Technologies, Santa Clara, CA).

### 2.6 Statistics

Biostatistical comparisons were performed in GraphPad Prism software. Differences in treatment‐dependent changes between groups were evaluated by 2‐way ANOVA with repeated measures for linked samples from each participant (e.g., treatment x SARM1 inhibitor). Post hoc comparisons for column (agonist) and row (antagonist) effects were performed using Šídák's multiple comparisons test. A *p*‐value less than 0.05 was considered significant, with a *p* < 0.10 interpreted as a trending result. For 2‐way ANOVA, if there is no significant interaction term, significant individual effects of independent variables are presented. If the interaction was significant, it is presented in the figures.

## 3 Results

### 3.1 *SARM1* Is Expressed in Human PBMCs

**FIGURE 1:**
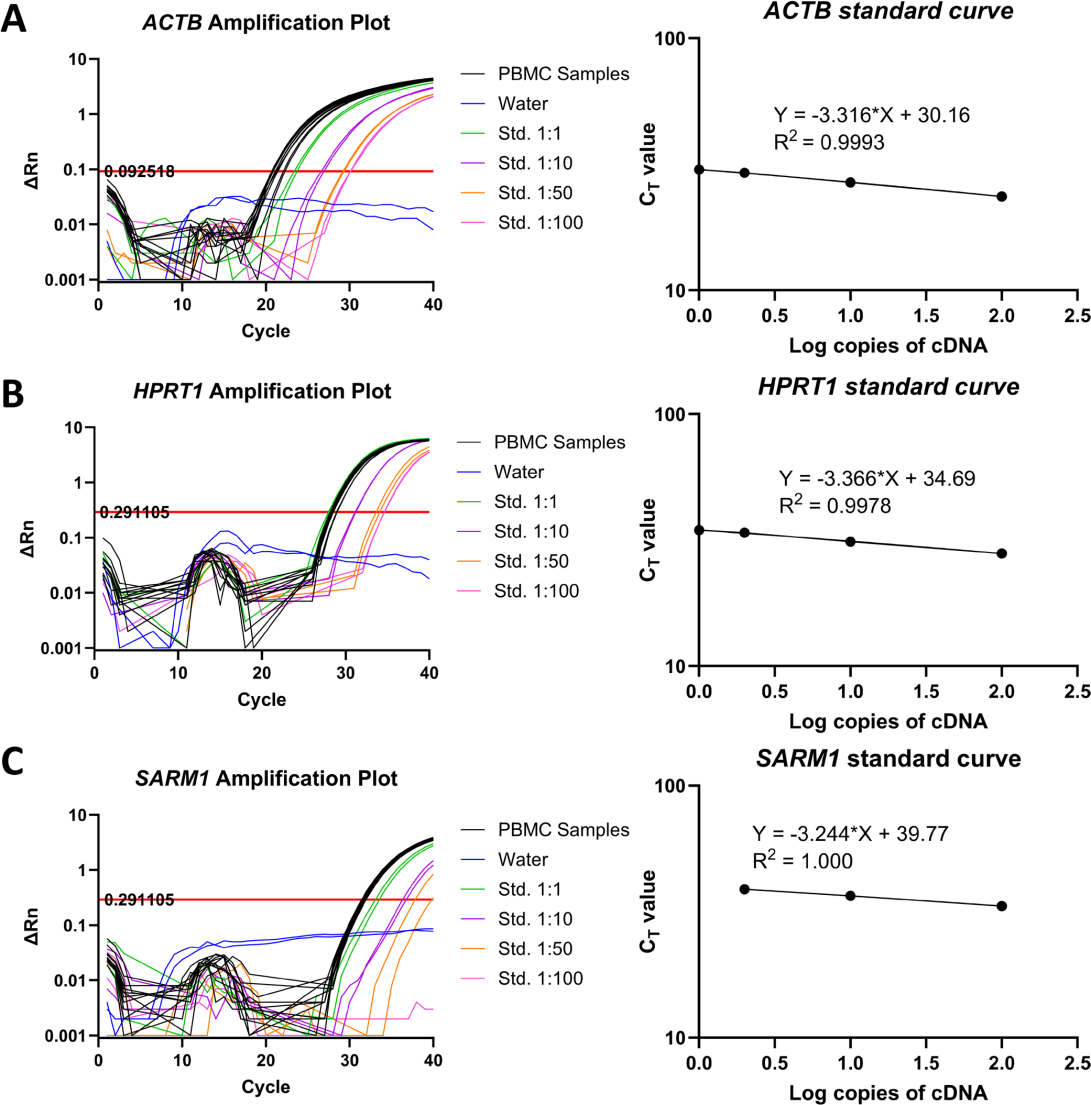
TaqMan qPCR amplification plots and standard curves of *SARM1* gene expression in human PBMCs. Gene expression was measured using TaqMan qPCR of human PBMC cDNA as well as a mix of cDNA for the standard curve. Amplification plots and standard curve plots are given for (A) *ACTB* expression, (B) *HPRT1* expression, and (C) *SARM1* expression. All samples and standard curves were run in duplicate and with *n* = 6 PBMC samples. The left‐side graphs have x‐axis denoting the C_T_ cycle and the y‐axis denoting change in normalized reporter (∆Rn) from the baseline signal. The Rn is calculated as the ratio of experimental fluorescence compared to the passive dye fluorescence (ROX), and the threshold (indicated by the red horizontal line) is determined as the level of fluorescence above background fluorescence. Right‐side graphs are log regressions of standard curve C_T_ outputs versus log cDNA concentrations.

*SARM1* gene expression in human PBMCs was measured with TaqMan qPCR. Amplification of *SARM1* based on the computed value threshold (C_T_) occurred after 31.7 ± 0.2 cycles with linear decreases in amplification observed during serial dilution of a standard curve sample (Figure 1). For comparison, C_T_ values for housekeeping genes *HRPT1* and *ACTB* were 28.4 ± 0.3 and 21.2 ± 0.5, respectively with linear changes in amplification observed during serial dilution (Figure 1). This reaffirms positive *SARM1* expression in PMBCs, as reported previously [7, 8, 9, 10, 11, 12, 13, 14, 15, 16, 17, 18, 19].

### 3.2 cADPR Is a Functional Readout of SARM1 Activity in PBMCs

**FIGURE 2:**
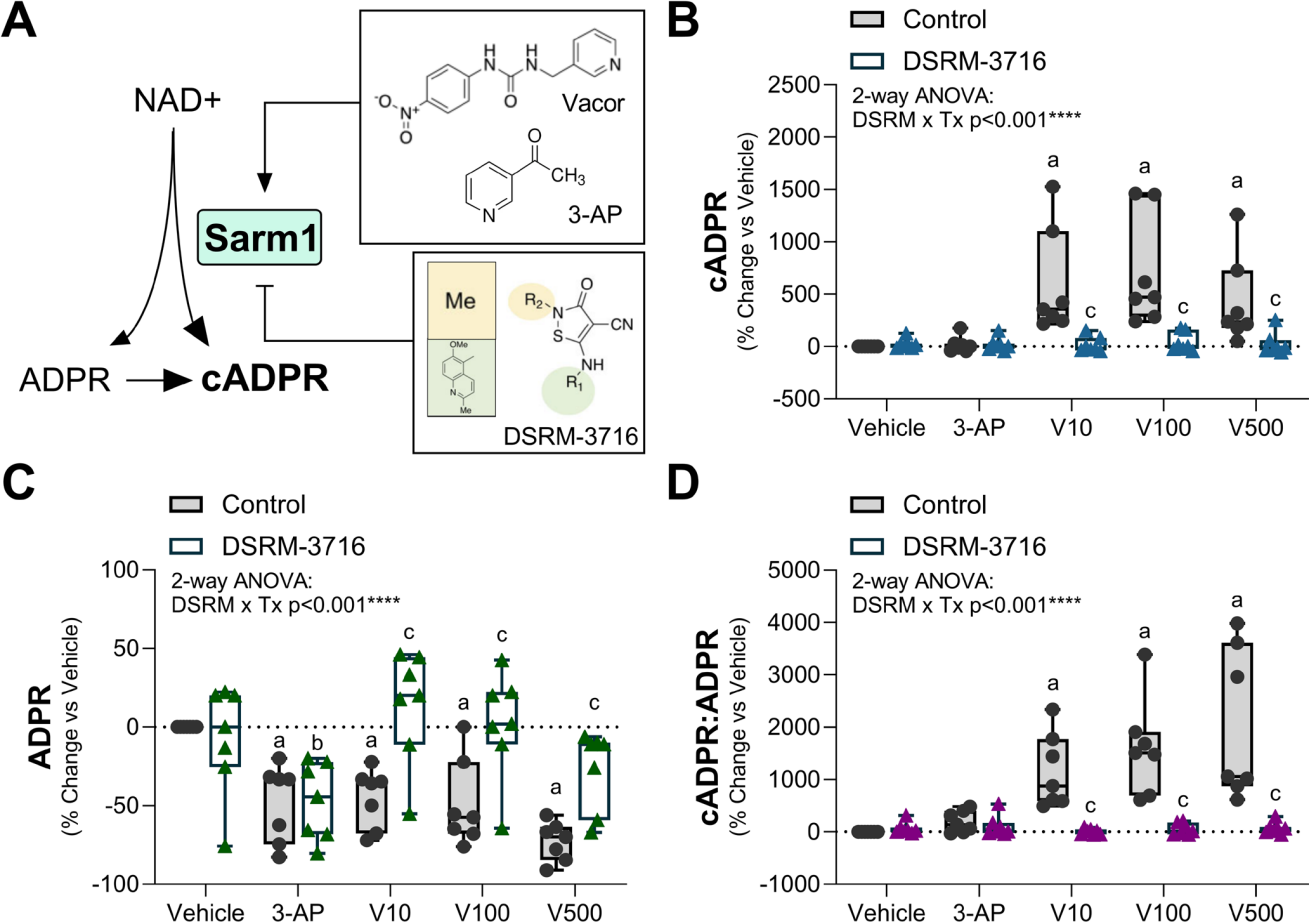
SARM1‐dependent elevation of cADPR in human PBMCs. PBMCs from each participant were stimulated with SARM1 agonists 3‐acetylpyridine (3‐AP; 1 mM) and pyrinuron, also known as Vacor (V; 10, 100, 500 μM), for 4 h to activate the enzymatic activity of SARM1. To test specificity for SARM1, a paired sample of cells within each group was simultaneously treated with 100 μM of SARM1 inhibitor DSRM‐3716. Responses are expressed as a percent change vs. each individual vehicle control. (A) Model of agonist and antagonist action on the SARM1 NADase enzyme. (B) Cyclic ADP‐ribose (cADPR). (C) ADP‐ribose (ADPR). (D) Ratio of cADPR:ADPR for each individual sample. 2‐way ANOVA with repeated measures for linked samples from each participant with Šídák's multiple comparisons test. ^a^ *p* < 0.05 vs. Vehicle Control. ^b^ *p* < 0.05 vs. Vehicle DSRM‐3716. ^c^ *p* < 0.05 vs. Control within the same treatment group.

Activation of SARM1 in neurons increases the cellular pool of cADPR (Figure 2A) [29, 31]. Consistent with this, cADPR was significantly elevated in PBMCs at even low concentrations of the SARM1 agonist Vacor (Figure 2B). Co‐administration with SARM1 inhibitor DSRM‐3716 prevented this increase, suggesting SARM1 dependence (Figure 2B). Unlike Vacor, SARM1 agonist 3‐AP had minimal effects on cADPR in PBMCs. The related byproduct ADP‐ribose (ADPR) was also regulated by Vacor in a SARM1‐dependent manner, with noted suppression after 4 h (Figure 2C).

To consider flux from ADPR to cADPR, we also quantified the ratio of cADPR:ADPR in our samples (Figure 2D). The cADPR:ADPR ratio was substantially elevated with Vacor treatment and restored to baseline by co‐administration of SARM1 inhibitor DSRM‐3716. Minimal elevations were also noted with 3‐AP, but this was not statistically significant. Overall, these findings show that SARM1 agonist Vacor potently and selectively stimulates the accumulation of cADPR. Our findings also suggest that utilization of the cADPR:ADPR ratio, in addition to cADPR alone, may hold value in tracking SARM1 activation in human PBMCs. Beyond this, it is important to note that treatment with DSRM‐3716 alone in the vehicle control group did not significantly alter cADPR, ADPR, or the cADPR:ADPR ratio, suggesting minimal basal SARM1 activity in freshly isolated PMBCs from healthy participants (Figure 2B–D).

### 3.3 SARM1 Agonists Deplete NAD+ and NMN With Differential Regulation of NaMN in PBMCs

When activated, the SARM1 NADase depletes cellular NAD+ and is regulated by metabolites upstream of NAD+ synthesis, including NMN and nicotinic acid mononucleotide (NaMN). Physiologically, high NMN:NAD+ activates SARM1. This is bypassed by direct chemical SARM1 activators 3‐AP and Vacor after their conversion to 3‐AP‐MN and Vacor‐MN and binding to the allosteric site [27, 28]. NaMN can also competitively bind the allosteric site of SARM1 and suppress NMN‐induced SARM1 activation [32].

**FIGURE 3:**
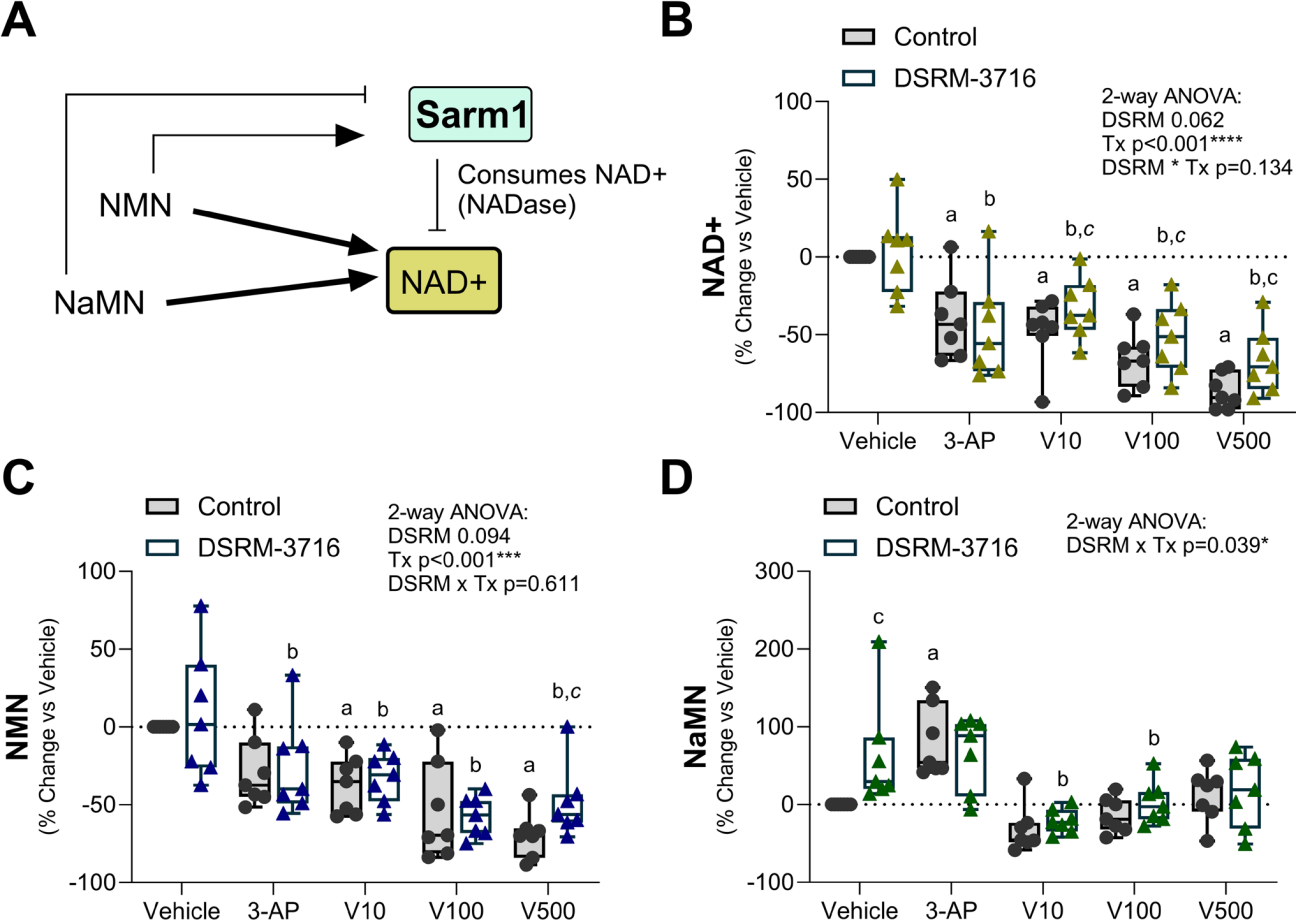
Generation and utilization of NAD+ after SARM1 activation in PBMCs. PBMCs from each participant were stimulated with SARM1 agonists 3‐acetylpyridine (3‐AP; 1 mM) and pyrinuron, also known as Vacor (V; 10, 100, 500 μM), for 4 h to activate the enzymatic activity of SARM1. To test specificity for SARM1, a paired sample of cells within each group was simultaneously treated with 100 μM of SARM1 inhibitor DSRM‐3716. Responses are expressed as a percent change vs. each individual vehicle control. (A) Model of nicotinamide adenine dinucleotide (NAD+) production and consumption by the SARM1 NADase enzyme. (B) NAD+. (C) Nicotinamide mononucleotide (NMN). High NMN:NAD+ activates SARM1, but this is bypassed by 3‐AP and Vacor which lead to direct activation of SARM1 independent of NMN. (D) Nicotinic acid mononucleotide (NaMN). High NaMN inhibits SARM1 and competes for binding of agonists 3‐AP (as 3‐AP‐MN) and Vacor (as VacorMN). 2‐way ANOVA with repeated measures for linked samples from each participant (*n* = 7) with Šídák's multiple comparisons test. ^a^p < 0.05 vs. Vehicle Control. ^b^p < 0.05 vs. Vehicle DSRM‐3716. ^c^p < 0.05 vs. Control within the same treatment group. ^*a,b,c*^Same comparisons with *p* < 0.1.

Treatment with SARM1 activators 3‐AP and Vacor significantly decreased NAD+ (Figure 3B). Partial restoration of NAD+ occurred with administration of SARM1 inhibitor DSRM‐3716 in the Vacor groups only. This suggests the occurrence of SARM1‐independent effects of both 3‐AP and Vacor on NAD+ biosynthesis in PBMCs, as will be discussed below. SARM1 agonists 3‐AP and Vacor also depleted NMN with partial restoration by DSRM‐3716 in the 500 μM Vacor group only. SARM1 agonist 3‐AP, but not Vacor, selectively elevated SARM1 inhibitor NaMN (Figure 3D). Elevated NaMN was also noted in the vehicle control group after treatment with SARM1 inhibitor DSRM‐3716.

### 3.4 Depletion of ATP and AMP Occurs at High Doses of Vacor

**FIGURE 4:**
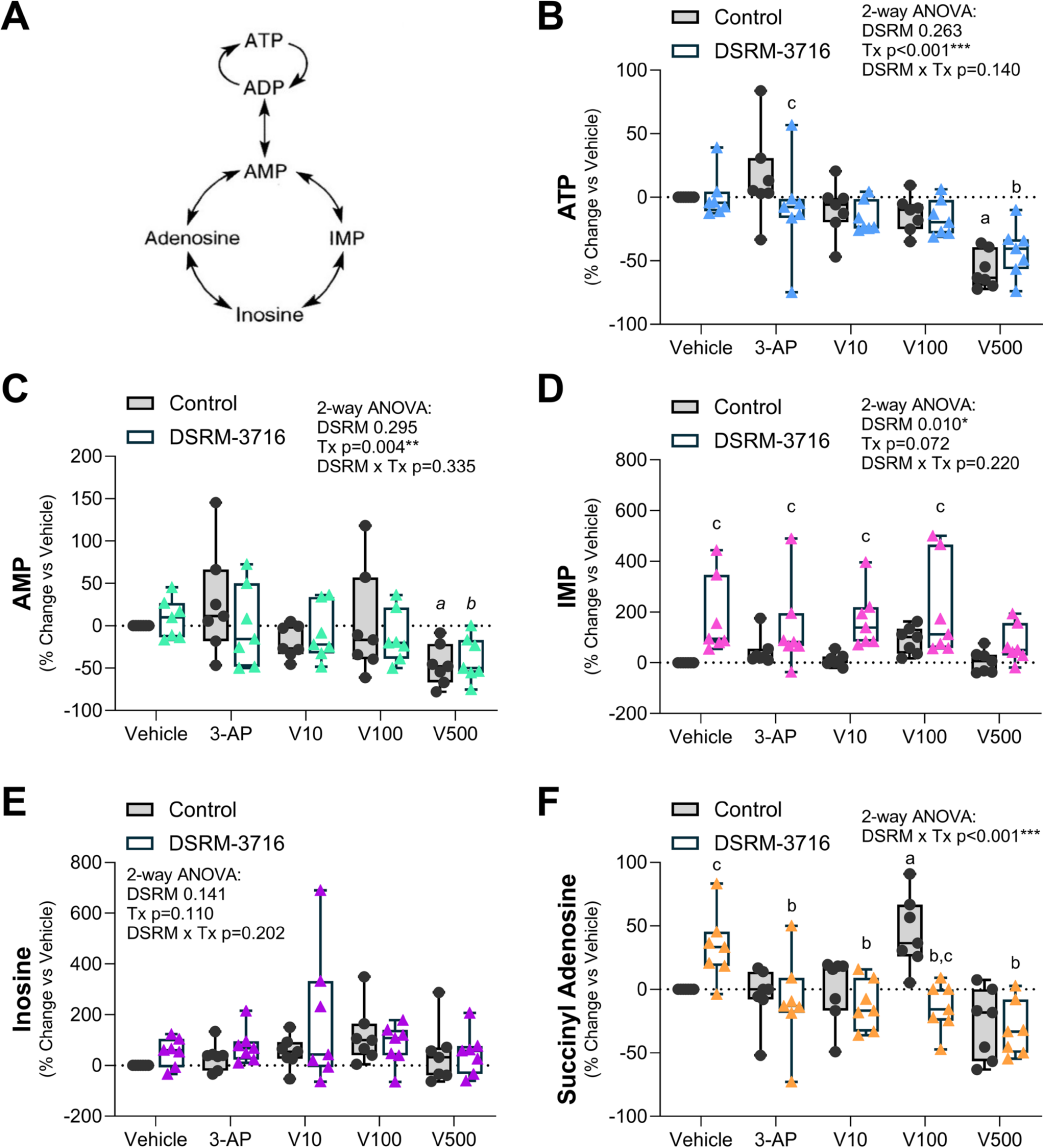
Tracking of ATP and downstream metabolites. PBMCs from each participant were stimulated with SARM1 agonists 3‐acetylpyridine (3‐AP; 1 mM) and pyrinuron, also known as Vacor (V; 10, 100, 500 μM), for 4‐h to activate the enzymatic activity of SARM1. To test specificity for SARM1, a paired sample of cells within each group was simultaneously treated with 100 μM of SARM1 inhibitor DSRM‐3716. Responses are expressed as a percent change vs. each individual vehicle control. (A) Model of ATP conversions and metabolic cycling. (B) ATP. (C) AMP. (D) IMP. (E) Inosine. (F) Succinyl adenosine. 2‐way ANOVA with repeated measures for linked samples from each participant (*n* = 7) with Šídák's multiple comparisons test. ^a^p < 0.05 vs. Vehicle Control. ^b^p < 0.05 vs. Vehicle DSRM‐3716. ^c^p < 0.05 vs. Control within the same treatment group. ^*a,b,c*^Same comparisons with *p* < 0.1.

We also examined changes in adenosine triphosphate (ATP) and adenosine monophosphate (AMP) by mass spec to track the health of the treated PBMCs (Figure 4A). Depletion of both ATP and AMP was observed in the high‐dose Vacor group only (500 μM), independent of SARM1 inhibition with DSRM‐3716 (Figure 4B,C). Clumping and deterioration of PBMCs in this group were also noted upon microscopic examination (data not shown), suggesting that loss of ATP and AMP was likely secondary to drug toxicity and cell death.

### 3.5 Elevation of IMP Occurs After Treatment With SARM1 Antagonist DSRM‐3716

AMP is converted to metabolites, including inosine monophosphate (IMP), inosine, and adenosine. Consistent elevation of the cellular IMP pool was observed after treatment with SARM1 inhibitor DSRM‐3716 across all groups (Figure 4D). There were no changes in cellular inosine (Figure 4E) and variable shifts in succinyl adenosine in the vehicle and 100 μM Vacor groups only (Figure 4F).

## 4 Discussion

### 4.1 Summary

Overall, our results indicate that human PBMCs have detectable expression of *SARM1* at the gene level. In addition, we show evidence of functional SARM1 enzymatic activity and accumulation of cADPR in PBMCs after treatment with the SARM1 agonist Vacor, which was blocked by the SARM1 inhibitor DSRM‐3716. In neurons, cADPR has previously been shown to be a readout of SARM1 NADase activity with high sensitivity and specificity [29]. This finding is of high interest because SARM1‐dependent elevations in cADPR occur prior to the onset of irreversible cell death and may serve as an early marker of reversible SARM1 activity, possibly allowing for early detection and treatment intervention. Of note, based on either cAPDR, ADPR, or the cADPR:ADPR ratio, SARM1 had limited basal activity in isolated PBMCs. This is similar to neurons, which have very low basal activity and suggests that clinical studies likely need to utilize both activators and inhibitors of SARM1 to reliably track SARM1 activation potential, in addition to monitoring for basal SARM1 activity, across different disease contexts [29].

### 4.2 Working Model of SARM1 Activation in PBMCs

**FIGURE 5:**
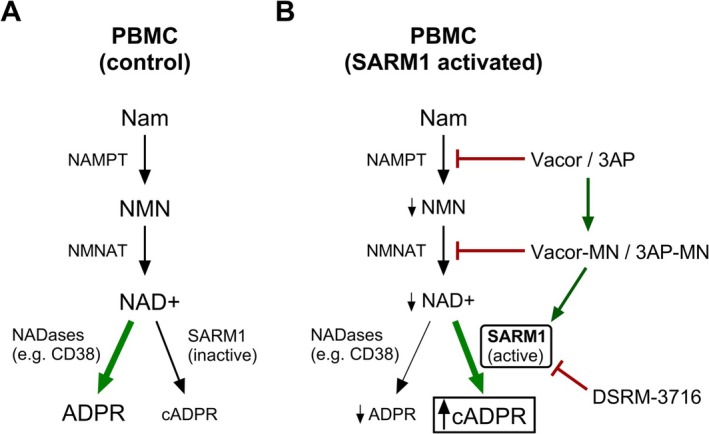
Working model of PBMC NAD+ metabolism and cADPR accumulation upon chemically induced SARM1 activation. Model for NAD+ metabolism in (A) control and (B) SARM1 activated PBMCs. The observation that SARM1 activators decrease NAD+ in a manner that is only partially SARM1‐dependent can be best explained if we consider (#1) the relatively low expression of *SARM1* in PBMCs relative to other NADases such as CD38 and (#2) the direct regulation of upstream NAD+ biosynthesis through the known actions of 3‐AP and Vacor on NAMPT and NMNAT. In native form, 3‐AP and Vacor compete with NAM as an alternative substrate for NAMPT. As observed in our data, this consequently decreases the generation of NMN. Biosynthesis of NAD+ is then limited by both a reduction in precursor NMN and direct inhibition of NMNAT by Vacor‐MN and 3‐AP‐MN. Vacor‐MN and 3‐AP‐MN also increase NAD+ consumption by activating SARM1. Addition of orthostatic SARM1 inhibitor DSRM‐3716 blocks NAD+ consumption by SARM1 but does not prevent the decrease in NAD+ biosynthesis, and we anticipate that this explains the only partial recovery of the PBMC NAD+ pool. Second, regarding the SARM1 agonist‐dependent depletion of ADPR, we propose a model by which most cellular ADPR in PBMCs is generated by other highly expressed hematopoietic NADases such as CD38. Activation of SARM1 in PBMCs appears to preferentially convert NAD+ to cADPR, as reported previously in neurons. We propose that the depletion of the NAD+ pool and SARM1‐dependent generation of cADPR limits the availability of substrate for alternative NADase‐based generation of ADPR, resulting in the observed decrease.

Altogether, these data inform a working model of SARM1 activation in PBMCs (Figure 5) that is slightly different than what has been reported for neurons as it relates to changes in NAD+ and ADPR [29]. First, the observation that SARM1 activators decrease NAD+ in a manner that is only partially SARM1‐dependent can be best explained if we consider (#1) the relatively low expression of *SARM1* in PBMCs and (#2) the direct regulation of upstream NAD+ biosynthesis through the known actions of 3‐AP and Vacor on NAMPT and nicotinamide mononucleotide adenylyltransferase (NMNAT). In native form, 3‐AP and Vacor compete with NAM as an alternative substrate for NAMPT [27, 28]. As observed in our data, this consequently decreases generation of NMN. Biosynthesis of NAD+ is then limited by both a reduction in precursor NMN and direct competition of NMNAT by Vacor‐MN and 3‐AP‐MN. Vacor‐MN and 3‐AP‐MN also increase NAD+ consumption by activating SARM1 [27, 28]. Addition of orthostatic SARM1 inhibitor DSRM‐3716 blocks NAD+ consumption by SARM1 but does not prevent the decrease in NAD+ biosynthesis, and we anticipate that this explains the only partial recovery of the PBMC NAD+ pool after treatment with orthostatic SARM1 inhibitor. Second, regarding the SARM1 agonist‐dependent depletion of ADPR, we propose a model by which most cellular ADPR in PBMCs is generated by other highly expressed hematopoietic NADases, including CD38 (Figure 5A). Previous work has shown that in HEK‐293 T cells, SARM1 produces cADPR from NADase activity at a more efficient rate than CD38 when activated, while CD38 NADase more often results in an ADPR byproduct [33]. In our own data as well, activation of SARM1 in PBMCs appears to preferentially convert NAD+ to cADPR, as reported previously in neurons [29]. We hypothesize that depletion of the NAD+ pool and SARM1‐dependent generation of cADPR limits the availability of substrate for alternative NADase‐based generation of ADPR, resulting in the observed decrease (Figure 5B). Future work will be needed to clarify this point.

### 4.3 Functions of SARM1 in PBMCs and Clinical Considerations

Though this study was not intended to elucidate the function of SARM1 in PBMCs, some discussion of current literature is warranted. Early research on hematopoietic SARM1 focused on the role of its TIR domain in myeloid‐lineage cells. Dimerization of TIR domains classically activates toll‐like receptor (TLR) signaling, triggering downstream cascades that initiate the innate immune response. SARM1 is unique because it can interact with TIR‐containing adaptor proteins to inhibit proinflammatory signaling pathways. Thus, SARM1 is the only adaptor protein known to negatively regulate the pro‐inflammatory response. Previous work has implicated SARM1 in the inhibition of both MyD88 and TRIF [21, 34] adaptor proteins, while some groups have shown that SARM1 is only capable of interacting with TRIF [7, 35]. One group has shown evidence that this candidate anti‐inflammatory function of SARM1 is independent of its NADase activity [17]. Additionally, SARM1 could be altering immune pathways downstream of adaptor protein activation, specifically inhibiting the NLRP3 inflammasome [17, 19], AIM2 inflammasome [16], and AP‐1 activation [21]. Multiple studies interrogating SARM1 localization in the cell during TLR stimulation reveal that SARM1 colocalizes to the mitochondria during pathogen stimulation [9, 12, 19]. In mice, this co‐localization has been linked with pyroptosis of the cell [12, 17, 19] as well as apoptosis specifically in T‐cells and NK cells [9].

Specific to the NADase activity of SARM1, there is emerging but limited evidence that SARM1 NADase activation may alter inflammatory cytokine production with two studies showing a SARM1‐mediated decrease and the other showing an increase. First, in THP‐1 cell lines with SARM1 overexpression, treatment with CZ‐48, a SARM1 activator, lowered the inflammatory response of the cells [17]. However, this study was done in a human cell line without detectable endogenous SARM1, and overexpression is less physiologically translatable. Additionally, CZ‐48 is an activator of SARM1, but it is also an inhibitor of the NADase CD38 [33] which could confound their conclusions. By contrast, a 2026 study demonstrated that synovial macrophages from patients with rheumatoid arthritis (RA) have high expression of *SARM1* and that a SARM1 inhibitor could limit consumption of NAD+ and production of cADPR in vitro and decrease the inflammatory profile of human synovitis chimeric mice in vivo [25]. This poses an application for SARM1 NADase‐dependent functionality in RA synovitis pathology. Similarly, a third study using THP‐1 cells in addition to mouse BMDMs showed that SARM1 expression was regulated by METTL1 and that SARM1 overexpression in BMDMs lacking METTL1 could decrease NAD+ to promote production of inflammatory cytokines [36]. The reason for the conflicting role of SARM1 as an activator or inhibitor of inflammation remains unclear. However, to advance the field, our study provides a model by which endogenous SARM1 NADase is present and can be activated in human primary blood cells, which can be applied to future work.

The collective relevance of these findings to human inflammation and immunity in vivo remains unclear, particularly since most prior studies have relied on artificial overexpression of SARM1 in immortalized cell lines or mouse macrophages. When considering the potential effects of pharmacologic SARM1 inhibitors on the hematopoietic system, two primary observations warrant further discussion. First, the TLR‐dependent actions of SARM1 are anticipated to be independent of the NADase activity of SARM1 through binding of the TIR domains. This means that any functions related to inflammasome activation and pathogen clearance may not be affected by pharmacologic inhibitors that selectively target the NADase site. Second, if SARM1 NADase is activated in human blood cells, there may be shifts in inflammatory cytokine production. However, it remains unclear at this point whether production would be increased or decreased and, furthermore, how this would balance with the candidate TLR‐modifying effects of the protein. Overall, future clinical trials may benefit from monitoring systemic biomarkers of inflammation, immune function, and SARM1 activation in PBMCs.

### 4.4 Limitations and Additional Considerations

We found that SARM1 agonist 3‐AP, which works well in neurons, did not fully activate SARM1 in PBMCs. This conclusion is based on the lack of changes in metabolite cADPR, which were readily observed with Vacor. One possible explanation for this is that 3‐AP selectively elevated the cellular pool of SARM1 inhibitor NaMN in PBMCs, which may have led to autoinhibition of the SARM1 enzyme. This may also be due to the low potency of 3‐AP‐MN relative to Vacor‐MN in binding to SARM1 [27, 28]. The second unexpected result was the consistent elevation of IMP by DSRM‐3716 regardless of treatment group. It may be that IMP can serve as a readout of SARM1 inhibition in PBMCs. However, the mechanisms driving these changes in NaMN and IMP in PBMCs remain unknown.

## 5 Conclusions

Altogether, we propose an assay for measuring SARM1 activation potential in human PBMCs by treating with optimal doses of SARM1 activator (100 μM Vacor) and inhibitor (100 μM DSRM‐3716). As in neurons, our data also reveal that both cADPR and the cADPR:ADPR ratio can be used as a primary readout for SARM1 activity in PBMCs. We anticipate that this same strategy can be applied to study SARM1 activity in any cell type of interest. Beyond this, we suggest quantifying NAD+ to monitor SARM1 NADase activity, NMN and NaMN to track the accumulation of SARM1 modulators, ATP/AMP as a readout of cellular health, and IMP as a putative marker of SARM1 inhibition.

## Author Contributions

**Lila F. Dabill:** data curation, formal analysis, conceptualization, investigation, writing – original draft, writing – review and editing. **Ivana R. Shen:** conceptualization, data curation, formal analysis, investigation, methodology, writing – review and editing. **Jennifer M. Brazill:** conceptualization, data curation, formal analysis, investigation, methodology, writing – review and editing. **Alicia Neiner:** data curation, formal analysis, validation, methodology, writing – review and editing. **Yo Sasaki:** conceptualization, data curation, formal analysis, resources, methodology, writing – original draft, writing – review and editing. **Erica L. Scheller:** conceptualization, formal analysis, funding acquisition, resources, investigation, project administration, supervision, visualization, writing – original draft, writing – review and editing.

## Funding

This work was supported by HHS | National Institutes of Health (NIH), U24‐DK115255.

## Conflicts of Interest

The authors declare no conflicts of interest.

## Data Availability

The data that support the findings of this study are available in the Materials and Methods and Results of this article.
